# Editorial: Metabolic Regulation of Diatoms and Other Chromalveolates

**DOI:** 10.3389/fpls.2022.897639

**Published:** 2022-05-03

**Authors:** Justine Marchand, Hanhua Hu, Kalina Manoylov, Benoît Schoefs

**Affiliations:** ^1^Metabolism, Molecular Engineering of Microalgae and Applications, Laboratory Biologie des Organismes, Stress, Santé Environnement, IUML – FR 3473 CNRS, Le Mans University, Le Mans, France; ^2^Key Laboratory of Algal Biology, Institute of Hydrobiology, Chinese Academy of Sciences, Wuhan, China; ^3^Department of Biological and Environmental Sciences, Georgia College & State University, Milledgeville, GA, United States

**Keywords:** microalgae, stress, omics, physiology, carbon metabolism

Microalgae are amongst the most abundant aquatic organisms. Microalgae form a polyphyletic group of organisms and diatoms belong to the Heterokonta clade (Kroth, 2015). This phylum evolved as a result of complex endosymbiosis and horizontal gene transfers from (cyano)bacteria and other microorganisms, including fungi [e.g., Thiriet-Rupert et al. (2016)], conferring them with unique biological features like efficient sequestering of dissolved CO_2_, emitting a significant part of the oxygen (Benoiston et al., 2017), and performing efficient metabolic reorientation. Last but not the least, diatoms need silicon to build their cell wall by means of a network of nano-patterns forming very aesthetic decorations. To this end, diatoms rendered and still render enormous ecosystem services contributing significantly to several of the biogeochemical cycles and to the establishment of most of ocean food chains (Benoiston et al., 2017). Indeed, diatoms colonized successfully a wide range of environments, including the narrowest niches [e.g., Schoefs et al. (2020)] thanks to a very diversified and original metabolism [e.g., Allen et al. (2011)] and a high capacity to regulate it in order to acclimate to particular conditions [e.g., Heydarizadeh et al. (2017)]. The overload of these protective mechanisms results in cell death, making diatoms interesting organisms for the assessment of water quality (Szczepocka et al., 2021). In addition, microalgae have a huge potential for biotechnological applications (Sharma et al., 2021). However, biotechnology based on microalgae remains in its infancy and its development depends on the resolution of several bottlenecks (Vinayak et al., 2015) about which this theme takes stock:

-*A deeper knowledge of the basic cellular mechanisms*: Being photosynthetic organisms, diatoms convert sunlight energy into chemical energy used for running the Calvin-Benson-Basham (CBB) cycle along which CO_2_ is fixed and converted into triose phosphates, ultimately used as building blocks for the synthesis of all the other cellular compounds. If the step succession of the CBB cycle is well-established, the regulation pathways at the transcriptional and post-transcriptional levels remain less clear. The article by Launay et al. takes stock of the different regulation levels (i.e., gene transcription, proteins production and enzyme activity). Interestingly, the redox regulation of the metabolic enzymes appears less important in diatoms than in green algae whereas the regulation at the transcriptional level seems to be widespread. The review also suggests that the role of post-translational modifications has been so far overlooked and needs further investigations. The contribution by Xie et al. on N-glycosylation in *Phaeodactylum tricornutum* Bohlin fills partially the gap. Using N-glycoproteomic and N-glycomic approaches, not less than 639 N-glycoproteins have been identified on the basis of 863 different N-glycopeptides.
-To feed efficiently the CBB cycle with CO_2_, diatoms import a considerable amount of CO_2_ thanks to the carbon concentration mechanisms (CCMs). Two CCMs, namely the biophysical pathway and the biochemical pathway, have been recognized so far (Clement et al., 2017) but on the basis of the few taxa investigated [*P. tricornutum*: Kroth et al. (2008); *Thalassiosira pseudonana* (Hustedt) Hasle et Heimdal CCMP 1335: Kustka et al. (2014), Tanaka et al. (2014); *T. pseudonana* Hasle & Heim. strain CCAP 1085/12: Clement et al. (2016); *T. weissflogii* (Grunow) Fryxell et Hasle CCMP 1336 [current name *Conticribra weissflogii* (Grunow) Stachura-Such. & D.M. Williams]: Reinfelder et al. (2000, 2004); (Roberts et al., 2007)], it was concluded that only the biophysical pathway is commonly active in diatoms (Kroth, 2015). The molecular data generated along the Tara Oceans expeditions (Bork et al., 2015) allowed Pierella Karlusich et al. to extend this view to other diatoms. The triose phosphates generated along the CBB cycle are partly stored in storage polysaccharides with either α- or β-glucosidic linked glucan polymers, namely glycogen/starch or chrysolaminarins/paramylon, respectively. *In silico* analyses of genomics data allowed the identification of candidates coding new enzymes involved in storage polysaccharide biosynthetic pathways and the reconstitution of the evolutionary history of the distribution of these pathways in Stramenopiles (Chabi et al.).
- *In-depth knowledge of the mechanisms regulating the response to individual or combined stresses:* Living in a complex environment, like the ocean, is not easy because of the frequent, and often significant, variations of the environmental factors, which can have additive effects. Scarsini et al. used a multidisciplinary approach to investigate the metabolic reorientation induced by the transition from nitrogen-replete to nitrogen starvation conditions in the marine diatom *P. tricornutum* cultured in a turbidostat. The switch between the two equilibria is driven by the intracellular nitrogen availability and mostly involves intracellular carbon reutilization rather than *de novo* carbon fixation. Nevertheless, chloroplast is kept in a stand-by mode allowing a fast resuming upon nitrogen repletion. The reutilization of the carbon involves several catabolic pathways including that of branched amino acids (Pan et al., 2017). In this theme issue, Pan et al. compiled omics data for providing a broad view on the contribution of amino acids to TAG accumulation. In another publication of this theme issue, Thangaraj et al. studied the effects of stress combination (temperature and silica) on the marine diatom *Skeletonema dohrnii* Sarno & Kooistra. The study found evidence for specific mechanisms to cope with these conditions: at low temperature, carbon and cell lipid quotas were higher while phosphate assimilation was reduced. This contrasts with silicate-limited cells in which phosphate cell quota was high while that of nitrate was low. Proteins associated with carbon fixation and photorespiration were downregulated in both stress conditions, while the genes coding proteins involved in carbohydrate and lipid syntheses were upregulated, confirming that lipid accumulation in stressed diatoms constitutes a default response mechanism as proposed by Heydarizadeh et al. (2019).
- The biochemical and physiological responses to stress rely on modifications of the transcription patterns. The diversity of experimental conditions, including taxon, growth and stress conditions, although providing complementary data, often prevents the determination of common modules in the responses to different stresses. Ait-Mohamed et al. analyzed RNAseq datasets generated under varying stress using Weighted Gene Correlation Network Analysis and identified 28 modules of co-expressed genes that reveal the fundamental principles on which co-regulation of genes expression in *P. tricornutum* relies.
- *The obtention of efficient biomolecule production platforms:* Despite the recognition that microalgae, including diatoms, synthesize many molecules of interest [e.g., Mimouni et al. (2012)] and the availability of tools for the genetic improvement of certain taxa (George et al., 2020), only a handful of diatom taxa are used on an industrial scale for the production of biomolecules. As pointed out by Vinayak et al. (2015), the biotechnological processes based on microalgae would benefit from a deeper knowledge in the basic functioning of diatoms coupled to a wider use of the biodiversity. The article by Galas et al. and Chuberre et al. compares the main morphotypes of *P. tricornutum* from the cell organization and metabolism point of views. The studies reveal that despite a common cell organization the oval cells exhibit a unique metabolic signature and excrete proteins more rapidly than the other morphotypes, probably due to specific activation of the secretory machinery. This characteristic could be helpful for improving the efficiency of non-conventional downstream processes such as biocompatible extraction (Gateau et al., 2021).
- *Cost effective methods for biomolecule quantification:* The utilization of proxies, such as the optical density at 750 nm for the cell density, are often used to follow and characterize biological phenomena. Several proxies are based on extraction of biomolecules and a few are available for the characterization of cell processes and cellular quota. In this theme issue, Scarsini et al. established a Fourier Transform InfraRed (FTIR) microscopy method for the simultaneous quantification of lipids, carbohydrates, and proteins in diatoms. The limits of the method have been estimated and the means to circumvent them are proposed.

## Conclusions

**Figure 1:**
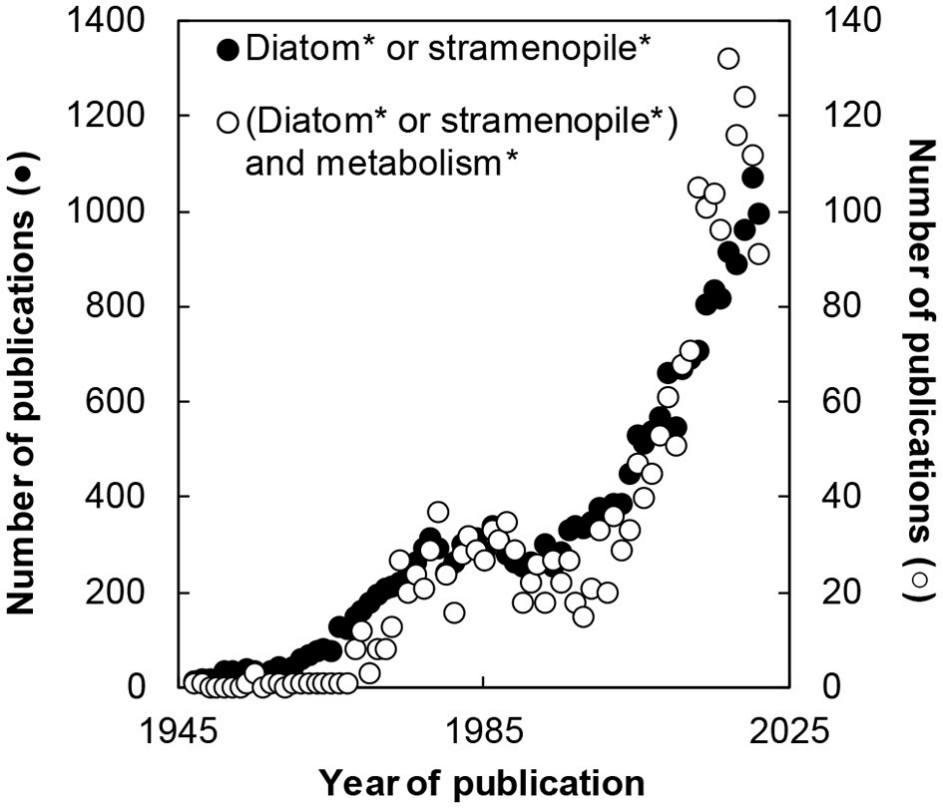
Progression of the number of publications on diatom's metabolism.

The research on diatoms and Stramenopiles is very dynamic. Since the World War II, more than 24,000 publications have been published with a title containing either word. *Circa* 10% of these articles are dedicated to their metabolism (Figure 1). This theme issue groups 11 articles describing the most recent research on the topic. These new data have been nicely welcomed by the scientific community with more than 30,000 views (https://www.frontiersin.org/research-topics/11978/metabolic-regulation-of-diatoms-and-other-chromalveolates - consulted on 2022 04 06) and 15 citations (WOS, all database, consulted on 2022 03 06).

## Author Contributions

KM, JM, HH, and BS contributed equally to the writing and editing of the editorial. All authors contributed to the article and approved the submitted version.

## Conflict of Interest

The authors declare that the research was conducted in the absence of any commercial or financial relationships that could be construed as a potential conflict of interest.

## Publisher's Note

All claims expressed in this article are solely those of the authors and do not necessarily represent those of their affiliated organizations, or those of the publisher, the editors and the reviewers. Any product that may be evaluated in this article, or claim that may be made by its manufacturer, is not guaranteed or endorsed by the publisher.
